# St. Mary's Hospital

**Published:** 1907-11-02

**Authors:** 


### HOSPITAL ADMINISTRATION.

### CONSTRUCTION AND ECONOMICS.

### ST. MARY'S HOSPITAL.

A GREAT OPPORTUNITY.

In one respect, namely in its financial position, St. Mary's Hospital appears at the moment to be in difficulties. When the Clarence Memorial Wing was first projected, many supporters of St. Mary's Hospital had grave doubts as to the wisdom of the proposed course, on the ground that if the new buildings were completed in their entirety there would be no funds to furnish, equip, and maintain them for the treatment of patients. Owing to the enthusiasm of one or two of the more active Governors at the time, it was determined to finish these buildings without first considering and securing the necessary revenue to sustain them in efficiency. It was found, as the buildings proceeded, that structurally to finish the new wing, it was necessary to sell out in the years 1904-1905 £38,000 of the hospital's investments. Further, in order to equip and furnish the wards, and to effect certain adjustments of the old building to the new, so as to enable the whole institution to be worked as one complete hospital, £12,500 more would be required. We thus arrive at a total of £50,500, and if St. Mary's Hospital had this sum invested, about half the necessary income for the maintenance of the Clarence Wing would be available. Whatever may have been the mistakes of the past, the present managers, by the excellent work they do for the public, are entitled to general sympathy, and we think the Committee would be wise to take steps to organise an effective appeal to raise this £50,500, in order to repair the shaken financial foundation of St. Mary's Hospital, and to put the new wards in a position to be opened to the sick poor.

£50,500 is, however, a large sum to collect for any hospital in times like the present. It is not reasonable to hope that so large an amount will be forthcoming, in the whole, for several years to come. Meanwhile the Clarence Wing remains unoccupied, and so tends to deteriorate in many ways, whilst it must, under the most economical conditions, be a considerable burden for necessary upkeep to the general resources of this institution. Such is the position at the moment, and it is one which calls for the exercise of courage and energy. By the exhibition of business enterprise, the immediate opening and occupation by patients of the whole of the Clarence Wing may, however, be secured. How can so desirable a result be accomplished in the readiest and speediest manner?

The history of St. Thomas's Hospital, where, in similar circumstances, the Governors had the courage to set aside one wing of the new buildings for paying patients, and of Guy's Hospital, which provides accommodation for paying patients at reasonable but inclusive rates, seems to us to indicate the wisest course which the authorities of St. Mary's Hospital can follow at the moment. Either the Clarence Wing in its entirety, or some other portion of the hospital which could be cleared of patients by their transfer to that wing, should be set aside, under proper conditions, for the accommodation of paying patients. No one who has a knowledge of the requirements of Londoners in the day of sickness can doubt, that there is a great and ever-increasing class of patients, who have no means in their homes for adequate treatment when sick, although they can afford the actual cost of their maintenance during illness, and something beyond this sum. St. Mary's Hospital would win for itself much popularity, show economical wisdom, re-establish the credit of its management, and do an infinity of good to many deserving people, if the Committee would energetically set to work and propound a scheme for the admission of paying patients into those portions of the buildings which are unoccupied for want of funds.

Will the authorities of St. Mary's Hospital have the wisdom to take advantage of this great opportunity? Mr. Harben, the chairman, is one of the most energetic and able of hospital chairmen; he is also an excellent man of business, who has shown commendable courage on more than one occasion, and we look to him to grasp the situation, and to solve the present difficulties of St. Mary's Hospital by establishing a system of paying wards. If this step is taken we make bold to say that the public will welcome it warmly, that their sympathies will go out in great measure to the authorities of St. Mary's Hospital, and that in a very short time, if paying rooms are opened, much or all of the £50,500 will be subscribed in order to give this institution the security of a reserve fund which it sadly needs at the moment. There are really no difficulties which cannot be overcome in making a change of this kind, because the rights of the medical profession can readily be safeguarded by allowing each patient in a paying ward to be attended, if he so wish, by his own doctor, and by setting aside a percentage of the patients' contributions to defray the cost of medical attendance. Criticism there possibly may be, because all new departures in connection with a particular institution must expect criticism; but this fact should inspirit and not depress the managers, for, if it is wisely utilised, it can only tend to add to the popularity and strength of St. Mary's Hospital. Mr. Harben thus has a splendid opportunity to win the gratitude of the public, and to bring to St. Mary's Hospital the active support, which it so greatly needs at present.

